# Biological Properties and Genetic Characterization of Novel Low Pathogenic H7N3 Avian Influenza Viruses Isolated from Mallard Ducks in the Caspian Region, Dagestan, Russia

**DOI:** 10.3390/microorganisms9040864

**Published:** 2021-04-17

**Authors:** Marina Gulyaeva, Maria Alessandra De Marco, Ganna Kovalenko, Eric Bortz, Tatiana Murashkina, Kseniya Yurchenko, Marzia Facchini, Mauro Delogu, Ivan Sobolev, Alimurad Gadzhiev, Kirill Sharshov, Alexander Shestopalov

**Affiliations:** 1 Federal State Budget Scientific Institution, Federal Research Center of Fundamental and Translational Medicine, 630117 Novosibirsk, Russia; murashka.djigurda@gmail.com (T.M.); xenia7yurchenko@gmail.com (K.Y.); sobolev_i@hotmail.com (I.S.); sharshov@yandex.ru (K.S.); shestopalov2@mail.ru (A.S.); 2 Department of Natural Science, Novosibirsk State University, 630090 Novosibirsk, Russia; 3 ISPRA Institute for Environmental Protection and Research, 40064 Ozzano dell’Emilia (BO), Italy; mariaalessandra.demarco@isprambiente.it; 4 Department of Biological Sciences, University of Alaska, Anchorage, AK 99508, USA; anna31kovalenko@gmail.com (G.K.); ebortz@alaska.edu (E.B.); 5 Department of Infectious Diseases, Istituto Superiore di Sanità, 00161 Rome, Italy; marzia.facchini@iss.it; 6 Department of Veterinary Medical Sciences, University of Bologna, 40064 Ozzano dell’Emilia (BO), Italy; mauro.delogu@unibo.it; 7 Department of Ecology and Sustainable Development, Dagestan State University, 367000 Dagestan, Russia; ecodgu@gmail.com

**Keywords:** avian influenza virus, H7N3, Caspian region, wild waterfowl

## Abstract

Avian influenza viruses (AIVs) are maintained in wild bird reservoirs, particularly in mallard ducks and other waterfowl. Novel evolutionary lineages of AIV that arise through genetic drift or reassortment can spread with wild bird migrations to new regions, infect a wide variety of resident bird species, and spillover to domestic poultry. The vast continental reservoir of AIVs in Eurasia harbors a wide diversity of influenza subtypes, including both highly pathogenic (HP) and low pathogenic (LP) H7 AIV. The Caspian Sea region is positioned at the intersection of major migratory flyways connecting Central Asia, Europe, the Black and Mediterranean Sea regions and Africa and holds a rich wetland and avian ecology. To understand genetic reservoirs present in the Caspian Sea region, we collected 559 cloacal swabs from *Anseriformes* and other species during the annual autumn migration periods in 2017 and 2018. We isolated two novel H7N3 LPAIV from mallard ducks whose H7 hemagglutinin (HA) gene was phylogenetically related to contemporaneous strains from distant Mongolia, and more closely Georgia and Ukraine, and predated the spread of this H7 LPAIV sublineage into East Asia in 2019. The N3 neuraminidase gene and internal genes were prototypical of AIV widely dispersed in wild bird reservoirs sampled along flyways connected to the Caspian region. The polymerase and nucleoprotein segments clustered with contemporaneous H5 HPAI (clade 2.3.4.4b) isolates, suggesting the wide dispersal of H7 LPAIV and the potential of this subtype for reassortment. These findings highlight the need for deeper surveillance of AIV in wild birds to better understand the extent of infection spread and evolution along spatial and temporal flyways in Eurasia.

## 1. Introduction

Currently, sixteen hemagglutinin (HA, from H1 to H16) and nine neuraminidase (NA, from N1 to N9) subtypes of avian influenza viruses (AIVs) have been isolated in a wide variety of bird species, often playing different epidemiological roles in the avian influenza (AI) spread. As was previously shown, wild waterfowl of the orders *Anseriformes* (ducks, geese, and swans) and *Charadriiformes* (gulls and shorebirds) form the natural reservoir of AIV, whose gene pool underlies the origin of other avian and mammalian influenza A viruses (IAVs) [1]. Wild ducks, in particular mallards (*Anas platyrhynchos*), have been recognized as key reservoir hosts, in which most AIV subtypes are maintained by migratory and/or resident bird populations [2]. The long-distance spread of AIV in nature is intrinsically linked with the movements and migratory behaviors of birds. It is supposed that bird movements are minimally affected by low pathogenic (LP) AIV infection, consequently resulting in the potential for virus shedding, spread, and reassortment during stopovers along the migration route [3]. The precise role of migrating and resident birds in amplifying and dispersing AIV is poorly understood, and in some occurrences, migratory birds can reinforce local circulation of AIV in resident populations [4,5]. Shifts from intraspecies to interspecies transmission and gene reassortment events represent additional aspects of AIV’s evolution strategy, enabling viral gene flow during the annual cycles of migratory behavior among wild bird species [6].

Some strains of LPAIV, belonging to H5 and H7 subtypes, are capable of mutating to highly pathogenic (HP) variants that can cause high rates of disease and death in poultry flocks. Highly pathogenic avian influenza (HPAI)—formerly termed fowl plague—has led to enormous socio-economic losses in the poultry industry and can result in fatal human infections. Recurrent zoonotic infections of H5 and H7 AIV have occurred in humans over the last two decades. In particular, bird-to-human transmissions are related to (i) the HPAIV H5N1 A/goose/Guangdong/1996 lineage (Gs/GD), which emerged in poultry in 1997 and then evolved into diversified HPAI H5 lineages; (ii) the more recent emergence of the LPAI H7N9 virus, which is currently co-circulating with HPAI H7N9 variants in poultry flocks [7]. While H5 subtypes remain infamous, the number of human infections with H7N9 viruses has far surpassed those attributed to H5 AIV [8]. Given that the endemic circulation of AIV subtypes in domestic poultry increases the probability of zoonotic transmission to humans, much recent attention has been focused on H7 AIV, particularly represented by the H7N9 subtype in China that has become established in domestic poultry and repeatedly transmitted to humans since 2013 [9]. Influenza A(H7N3) and A(H7N7) viruses have also been the causative agent in historical poultry and human infections in European countries and the Americas [10].

Unlike what happened with H5 emergence, the global transmission of H7N9 viruses through migratory birds has not been detected as yet [7]. However, a novel H7N3 reassortment originating from the zoonotic H7N9 virus was found in poultry meat illegally imported from China into Japan. The novel H7N3 virus was shown to efficiently replicate in chickens and mallards without lethal effect in the latter bird species, whereas previous zoonotic H7N9 subtypes were not well adapted to ducks. Based on these findings, this new H7N3 AIV might become established in wild bird populations and spread to remote areas through migratory routes [11].

Considering the emergence of HPAI subtypes, studies on AIV ecology in natural ecosystems are of particular importance to better identify emerging health risks associated with wild bird reservoir hosts. In this regard, the Caspian region is one of the key points for AIV surveillance in Eurasia, being located at the intersection of three major migratory routes: the Central Asian flyway, the East Africa/West Asia flyway and the Black Sea/Mediterranean flyway. Main migratory pathways from Europe, Africa and Asia cross here [12,13]. Due to the richness of large saltwater bays, lagoons, river deltas and lake systems, the western coast of the Caspian Sea represents a highly favorable route for the seasonal migrations of many bird species in Eurasia [14]. Ecological characteristics of these unique habitats allow wild waterfowl and shorebirds to use numerous Caspian wetlands for both wintering and nesting. Moreover, this region may also represent a possible stopover site for birds that migrate further to Europe or Africa. As stated above, the pathogenic and zoonotic potential of H7 strains makes them a substantial economic and public health concern. Furthermore, despite the health risk, it is unclear why specifically this virus became endemic in poultry and emerged in humans [10].

The significant increase of H7 zoonotic potential since 2013 and the genesis of highly pathogenic H7 underline the necessity of identifying and understanding the introduction of AIV into distinct geographic regions as well as the dynamics of AIV reassortment [8,15]. To understand AIV in the ecological context of the Caspian region, we conducted surveillance efforts in wild birds and poultry and analyzed sequence data from an evolutionary perspective. We isolated two new viruses of H7N3 subtype from mallard ducks (*A. platyrhynchos*), sampled on the western coast of the middle Caspian Sea (Republic of Dagestan, Russia). Although Caspian wetlands are the nesting, wintering and stopover grounds of a great number of migrating waterfowl, according to available literature, the isolation of the H7 AIV subtype seems to be a quite rare event in Caspian region in particular, and in Russia in general. The molecular and biological properties as well as the pathogenicity characteristics of these new AIV strains were analyzed in the present study.

## 2. Materials and Methods

### 2.1. Animal and Human Rights Statement

The work was performed in accordance with the ethical standards of laboratory animal treatment (Directive 2010/63/EU of the European Parliament and of the Council of 22 September 2010 on the protection of animals used for scientific purposes) and The Rules of Laboratory Practice in the Russian Federation (Order of the Ministry of Health of the Russian Federation No. 267 of 19 June 2003).

### 2.2. Sample Collection

Sampling was conducted during hunting seasons, from September 2017 to November 2018, in the territory of Papas Adzhi Lake, located fourteen kilometers from the Dagestan coastline (Caspian Sea region, Dagestan Republic, Russia). A total of 559 individual cloacal samples were collected from hunted wild birds belonging mostly (*n* = 429) to the Anseriformes order.

### 2.3. Influenza Virus Detection and Isolation

Standard protocols were used for virus isolation and further identification carried out in the BSL 3 Laboratory of the Federal Research Centre of Fundamental and Translational Medicine [16]. RNA was extracted from collected swabs using AmpliSens^®^ RIBO-sorb kit (InterLabService, Moscow, Russia). Reverse transcription was performed using Reverta-L kit (InterLabService, Moscow, Russia). Samples that tested PCR-positive for influenza A matrix (M) gene by “AmpliSens^®^ Influenza virus A/B-FL” Influenza virus A and Influenza virus B RNA detection kit (InterLabService, Moscow, Russia) were inoculated into 10-day-old embryonated chicken eggs. Allantoic fluid was collected 3 days post infection (dpi), processed and analyzed for influenza virus RNA via real-time PCR using the “AmpliSens ^®^ Influenza virus A-type-H5, H7, H9-FL” (InterLabService, Moscow, Russia) to reveal potentially dangerous subtypes, according to the manufacturer′s instructions.

### 2.4. Pathogenicity Tests in Chickens

The intravenous pathogenicity index (IVPI) test for A/mallard/Dagestan/1050/2018 and A/mallard/Dagestan/1051/2018 viruses was performed as described in the OIE Manual [17,18]. Ten 6-week-old specific-pathogen-free (SPF) chickens were intravenously (iv) inoculated with 0.1 mL of 1:10 diluted infective allantoic fluid (containing 10^6^ EID50 of the virus). The chickens were examined daily for clinical signs of the disease for 10 days. The pathogenicity index was calculated as the mean score per bird per observation. An index of 3 indicated that all birds died within 24 h; an index of 0 meant that no bird showed signs of illness during the 10-day observation period.

### 2.5. Experimental Infection of Mice

To evaluate the replication ability of A/mallard/Dagestan/1050/2018 and A/mallard/Dagestan/1051/2018 viruses in mice, forty 6-week-old BALB/c mice were divided into two groups of 20 each. Animals from each group were lightly anesthetized and intranasally inoculated with 10^6^ embryo infective doses (EID50) of virus in 50 µL of allantoic fluid. A group of negative control mice (*n* = 20) was inoculated intranasally with 50 µL of PBS. Mice were weighed and observed daily for 14 days post infection (d.p.i.). Three mice from each experimental group were euthanized 3, 5, 7 and 10 d.p.i., and lung tissue samples were aseptically collected for virus titration. Each tissue sample was homogenized in 1 mL of Eagle′s minimal essential medium containing the antibiotics penicillin and gentamicin and centrifuged at 4000 rpm for 5 min; the supernatant obtained was used to inoculate MDCK cells with an initial dilution of 1:10.

On 21 d.p.i., the remaining animals were euthanized, blood samples were collected, and serum was obtained. Serum samples were tested via HI assay for detection of antibodies to A/mallard/Dagestan/1050/2018 and A/mallard/Dagestan/1051/2018 viruses.

### 2.6. Sequencing

RNAs were isolated from allantoic fluid using a GeneJET viral DNA/RNA purification kit (Thermo Fisher Scientific) and treated with TURBO DNase (Thermo, Fisher Scientific). Up to 200 ng of RNA were used for the DNA libraries, which were prepared using a TruSeq RNA sample preparation kit, version 2 (Illumina, San Diego, CA, USA). Sequencing of the DNA libraries was conducted with a reagent kit, version 3 (600-cycle), on a MiSeq genome sequencer (Illumina) at SB RAS Genomics Core Facility (ICBFM SB RAS, Novosibirsk, Russia). Full-length genomes were assembled de novo with CLC Genomics Workbench, version 9.0 (Qiagen, Germantown, MD, USA).

Sequence accession numbers in GISAID (“global initiative on sharing avian flu data”) were as follows: EPI_ISL_331289 for A/mallard/Dagestan/1050/2018; EPI_ISL_331290 for A/mallard/Dagestan/1051/2018.

### 2.7. Molecular Characterization

#### 2.7.1. Amino Acid Analysis

Molecular characterization of amino acid sequences was performed to assess the presence of genetic markers potentially affecting biological characteristics of A/mallard/Dagestan/1050/2018 and A/mallard/Dagestan/1051/2018. Experimentally verified markers, known to be associated with AIV pathogenicity, host adaptation (e.g., receptor binding and replicative capacity) and transmission, were tested as described by Suttie et al. [19] and Mänz et al. [20]. Sequences examined in this study were first aligned with selected sequences available from GISAID using the CLUSTAL_X program [21] and then edited with BioEdit 7.05 [22]. NA and M2 genes of H7N3 isolates were also checked, as previously described, for the presence of known mutations associated with resistance towards neuraminidase inhibitors and adamantanes [23,24].

#### 2.7.2. Nucleotide Analysis

Nucleotide sequence identity analysis was performed with the Megalign program included in the DNASTAR Lasergene v.15 software. To perform a similarity search for high scoring sequence alignments between sequences under study and available sequence databases, NCBI and GISAID nucleotide BLAST were used.

#### 2.7.3. Phylogenetic Analyses

Available AIV sequences related to the initial H7N3 viruses in this study (A/mallard/Dagestan/1050/2018 and A/mallard/Dagestan/1051/2018) were searched and classified by segment using BLAST, and whole-genome sequences from influenza A viruses were downloaded from the Global Influenza Sharing Database (GISAID), EpiFlu and NCBI GenBank. Sequence alignments were constructed for each of the 8 gene segments using MAFFT [25], with manual correction in Geneious Prime 1 February 2020 software (https://www.geneious.com, accessed on 16 April 2021). Duplicate strains and viruses that had truncated sequences and had evidence of lab errors (assessed by root-to-tip divergence using TempEst) were removed, and a best-fitting root analysis for each dataset was determined using TempEst [26]. Sequences with significant recombination signals were excluded from the final data sets using RDP4 software [27].

#### 2.7.4. Maximum Likelihood Phylogenetic Analysis

The numbers of sequences used for the phylogenetic analysis are as follow: PB2 (*n* = 131), PB1 (*n* = 136), PA (*n* = 119), HA7 (*n* = 104), NP (*n*=105), NA3 (*n* = 116), MP (*n* = 119) and NS (*n* = 112). Phylogenetic trees for each gene segment were generated by the maximum likelihood (ML) method using the IQ-TREE [28] phylogenomic web-server by maximum likelihood with the ultrafast bootstrap (1000) branch supports [29]. For each gene segment the substitution model was determined using ModelFinder through the IQ-TREE [30], and the best-fit models according to BIC were used. The substitution models and the input data are indicated in Table A1. Phylogenetic trees were summarized, visualized and annotated in FigTree v1.4.4 (available online: http://tree.bio.ed.ac.uk/software/figtree/, accessed on 16 April 2021).

#### 2.7.5. Bayesian Phylogenetic Analysis

Additionally, temporal phylogeny was inferred for the HA genes separately using the time-scaled Bayesian approach and using MCMC available via the BEAST v1.10.4 package [31]. The total number of HA7 sequences used for the Bayesian phylogenetic analysis were 241 (*n* = 241). To look into the relationship between genetic divergence and time, we performed a root-to-tip regression analysis for a temporal signal using TempEst v.1.5 [26] by plotting the root-to-tip genetic distance estimated from the reconstructed 241 HA7 ML tree against the date of sampling. A strict molecular clock was used, with a coalescent GMRF Bayesian Skyride tree prior and a general-time reversible (GTR) model of nucleotide substitution with gamma-distributed rate variation among sites, with four categories and base frequencies estimated. The MCMC chain was run separately two times with 100 million iterations, with subsampling every 10,000 generations to assure convergence. From each chain, the first 10% of the samples was discarded as burn-in, and the runs for the HA7 segment were combined using the LogCombiner v1.10.4 program distributed as part of the BEAST package to generate a final posterior distribution tree that was used in subsequent analysis. Estimation of the ESS (Effective Sample Sizes) values was performed in Tracer v1.7.1 [32] as an evaluation criterion (ESS > 200 means that the current analysis yields a sufficient number of independent samples from the posterior distribution for interested parameters) for the proper convergence of each MCMC simulation of every posterior parameter. The maximum clade credibility (MCC) tree was generated and summarized using TreeAnnotator v1.10.4 with 10% burn-in. The tree was visualized, colored and annotated in FigTree v1.4.4 (available online: http://tree.bio.ed.ac.uk/software/figtree/, accessed on 16 April 2021).

## 3. Results

### 3.1. Isolation and Pathogenicity of Two Novel H7N3 LPAI Strains

To characterize AIV genotypes circulating in the Caspian region, cloacal samples were collected from hunted wild birds belonging mainly (*n* = 429/559) to the Anseriformes order. Sampling took place from September 2017 to November 2018, during hunting summer–autumn and winter–spring seasons, in the Papas Adzhi Lake region, located 14 km from the Dagestan coast of the Caspian Sea (Russia). Cloacal swabs collected from the hunted wild birds were tested for AIV by real-time PCR. Of 559 samples, 15 were positive for AIV (2.68%), all of which were collected from Anseriformes. Samples that tested positive for AIV were inoculated into 10-day-old embryonated chicken eggs. Allantoic fluid was collected three days post-infection and tested for the presence of hemagglutinin activity. Both viruses detected showed a titer of 1:32 HA units (HAU) per 50 μL in HA assay. Allantoic fluid was tested for H5, H7 and H9 subtypes, as required by the Russian Federal Service for Veterinary and Phytosanitary Surveillance, and two samples detected as H7 subtypes were used for IVPI testing and for further full genome sequencing. The novel viruses were named A/mallard/Dagestan/1050/2018(H7N3) and A/mallard/Dagestan/1051/2018(H7N3) and were submitted to GISAID.

No behavioral changes or disease manifestations were observed during the IVPI test in chickens inoculated with the two H7 viruses. Both isolates had IVPI scores of 0 and were not pathogenic for chickens [17]. The further study of novel viruses showed an LPAI pathotype based on the lack of polybasic cleavage sites in the HA gene, due to the ongoing genetic evolution occurring in AIV from Eurasia and Africa, frequently leading to reassortment events among LPAIV and HPAIV gene segments [5]. We also assessed the pathogenicity of A/mallard/Dagestan/1050/2018(H7N3) and A/mallard/Dagestan/1051/2018(H7N3) viruses in 6-week-old BALB/c mice. In both groups, mice did not lose weight or show other signs of disease. Active replication in lungs was not observed either by PCR or by infection of MDCK cells with respiratory turbinates. On 21 d.p.i., sera were taken and HI titers were found (Table S1). These results suggest that both viruses of the H7N3 subtype are nonpathogenic for mice and do not replicate effectively in mouse lungs. At the same time, strains reveal antigenic properties in mice models.

### 3.2. Nucleotide and Protein Analyses

The HA cleavage site pattern of both H7N3 strains showed the absence of multi-basic amino acids, which indicates that these viruses bear markers in the amino acid sequences (PELPKGR/GLF) of low pathogenicity (Table S2). Amino acid composition of the receptor-binding sites in the HA proteins was indicative of a preference for α 2,3-linked sialic acid receptors, which are predominant in avian species. Finally, five potential glycosylation sites were detected in the HA proteins (Table S2, Figure S1).

Both isolates had a full-length NA gene; no amino acid deletion of the stalk region of the NA protein associated with H7 adaptation to poultry was observed [33,34]. No mutations associated with resistance towards neuraminidase inhibitors and adamantanes—in NA and M2 genes, respectively—were observed in H7 isolates, indicating their susceptibility to these drugs. In detail, the N3 sialidase active sites were conserved; no changes in the catalytic (R118, D151, R152, R224, E276, R292, R371 and Y406–N2 numbering) and in the framework (E119, R156, W178, S179, D198, I222, E227, H274, E277, N294 and E425–N2 numbering) regions were detected.

Neither of the two H7 strains analyzed contained amino acid substitution in internal genes known to confer adaptation of AIV to mammalian hosts, such as E627K and D701N in PB2, nor specific mutations at residue 66 in PB1-F2 [19]. In particular, no deletion was observed within or at the C-terminal region of the NS1 genes, and both H7 strains had an ESEV amino acidic motif at the C-terminal region of this protein, which is typical of avian influenza viruses.

Both H7N3 isolates were also examined for amino acids, which have been previously shown to affect the biological characteristics of AIVs [19]. Several residues previously described in specific positions were observed (Additional Information 1). As verified by additional sequence alignment with other H7 strains from GISAID (data not shown), patterns observed in our H7N3 subtypes were present in most of H7 AIV, and among them only the 190S mutation in PA protein has been previously reported for the H7N3 subtype.

A/mallard/Dagestan/1050/2018 and A/mallard/Dagestan/1051/2018 nucleotide sequences were very similar when compared to each other (Figure S2), showing percent identities of 100.0% (PB2, PB1, NA and M gene segments), 99.9% (PA, HA, NP gene segments) and 99.7% (NS gene segment). As shown in Table S3, geographic distribution of NCBI and GISAID nucleotide BLAST results suggested that related AIV occurred in Asia (HA, PB1), Africa (NA, MP, NP) and Europe (PB2) or had different geographic and/or host origins (PA, NS).

### 3.3. Phylogenetic Analyses

To better understand the evolutionary relationships between the novel H7N3 AIV and other AIV strains, maximum likelihood (ML) and Bayesian phylogenies were constructed and analyzed. Overall, ML phylogenetic analyses suggested that the segments of the H7N3 AIV clustered geographically with Asian (HA, PB1, PA, NS), African (MP, NA, NP) and European (PB2) AIV lineages (Figure S3). Internal gene segments from the H7N3 AIV also clustered with circulating LPAI across Eurasia, including the Indian subcontinent, for example, as shown by close phylogenetic relationships of polymerase genes (PB2, PB1 and PA) and NS, MP, and NP with LPAI isolated ducks and teals in Bangladesh (Figure S3). Interestingly, the internal genes PB2, PB1, PA and NP of the H7N3 viruses also showed close relationships to those of the high pathogenicity AIV (HPAIV) H5N8, H5N6 and H5N5 from the Eurasian lineages (H5 clade 2.3.4.4b and derivatives). This indicates that the H7N3 viruses in this study shared an internal gene pool of viral segments found in both LPAI and HPAI in Eurasia, suggesting a propensity for diverse reassortment in host reservoirs.

**Figure A1:**
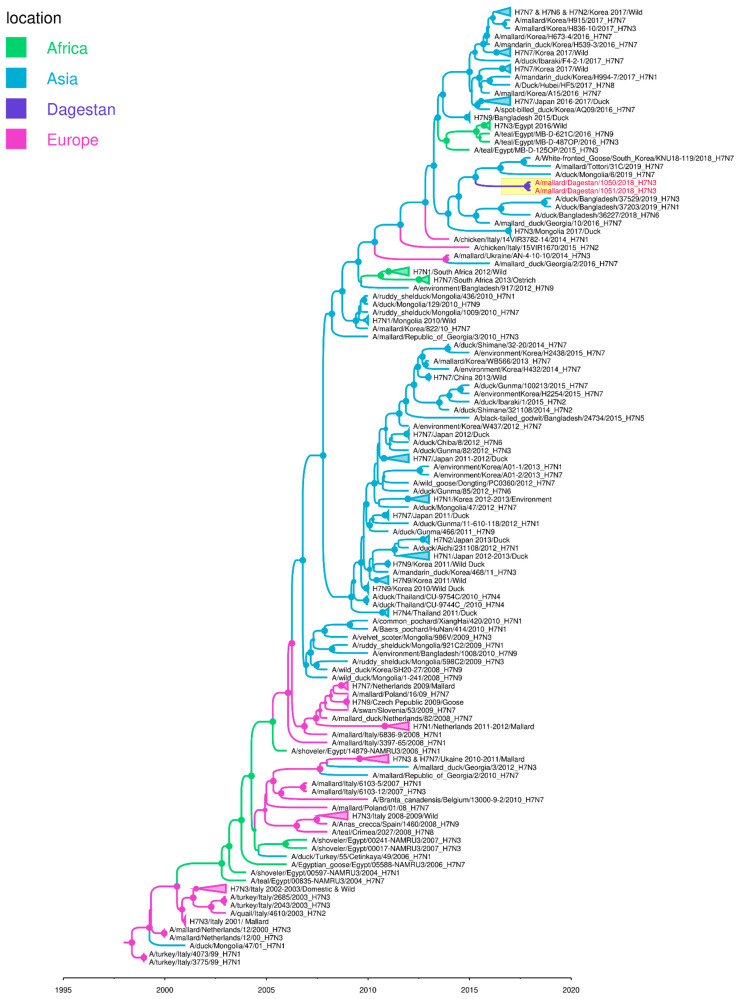
Time-scaled Bayesian maximum clade credibility analysis of H7 HA gene segment from novel H7N3 AIV isolated in Dagestan, Russia. A time-scaled MCC tree was constructed using Bayesian coalescence analysis in BEAST with a reference set of 241 H7 sequences as described in the methods section. Time and geographic origin of AIV genes are indicated by colors. Well-supported posterior probabilities (>0.65) of the nodes are indicated by circles. The size of the circles is proportional to the inferred posterior probabilities.

Root-to-tip regression analysis of the temporal signal from the 241 HA7 dataset revealed a strong temporal signal (a correlation coefficient of 0.97 and a coefficient of determination (R2) of 0.95) (Figure S4), suggesting positive correlations between genetic distances and sampling time. Bayesian coalescence analysis indicates that the H7 gene of the novel H7N3 viruses in this study (A/mallard/Dagestan/1050/2018 and A/mallard/Dagestan/1051/2018) are closely related and clustered to Asian wild bird-origin H7 LPAI viruses (Figure A1). It is noteworthy that the HA7 segment arrived from European domestic bird-origin viruses and is phylogenetically related to the A/chicken/Italy/14VIR3782-14/2014_H7N1 LPAIV isolate. The time-scaled maximum clade credibility (MCC) tree based on the strict clock was used to estimate the time of the most recent common ancestor (TMRCA) of the 241 H7 viruses analyzed on the tree. For these sequences, only the sampling year was provided and not the exact sampling dates. The estimated decimal year for the most recent common ancestor for the H7 gene compatible with the TMRCA is 1998.4, with the 95% credible interval 1997.9–1998.8. The estimated rate of evolution (substitutions per site per year) of the H7 gene of the H7N3 viruses showed the mean substitution rate—4.04 × 10^−3^ (with 95% HPD interval 3.63 × 10^−3^–4.45 × 10^−3^). This rate is similar to those previously described for avian influenza A viruses, where a mean substitution rate for HA gene was estimated as 3.92 × 10^−3^ [35]. Thus, lineages deriving from the ancestral H7 gene segment may have been in circulation for more than two decades in avian reservoirs in Eurasia, highlighting the persistence of this subtype in wild bird populations.

## 4. Discussion

In previous studies, LPAI AIV of the H7N3 subtype were isolated from numerous bird species [36]. Nearly all of these strains belong to classical avian-like lineages. In the present study, we identified two novel H7N3 LPAI isolates from wild mallard ducks; this is the first report of H7N3 LPAIV detection in the ecologically rich Caspian Sea region. Here, we characterized these two new isolates and revealed the possibility of ongoing exchange of gene segments with other Eurasian and African LPAI viruses, circulating mostly along the Black Sea/Mediterranean flyway. The constellation of H7, N3 and internal gene segments, although phylogenetically similar between the two viruses discovered in this study, actually represent a widely dispersed group of AIV gene segments found across wild bird flyways in Eurasia (Figure S3). For example, the H7 (LPAI) gene segment was similar to contemporary isolates from Georgia, Ukraine and Mongolia, and antecedent to H7 AIV found in East and South Asia in 2019 (Figure A1). Bayesian phylogenetic analysis indicated that the ancestor of this H7 gene segment may have been in circulation in Eurasia for two decades or more (Figure A1), suggesting the widespread dispersal in both time and space, and potentially multiple host bird species, of this subtype. Moreover, close relatives of the internal gene segments (polymerase and NP) have been found circulating H5 HPAI in Eurasia (clade 2.3.4.4b and derivatives), and all internal gene segments have phylogenetic relatives in LPAI sampled from Anatidae across Eurasia (Figure S3), highlighting the plasticity of the AIV reservoir and the need for much increased genetic surveillance of AIV and hosts along migratory flyways connected to the Caspian and Black Sea regions (Figure S3).

As previously reported by Hiono et al. (2015), H7 HA segments are phylogenetically divided into five main lineages: Historical European, Australian, Equine, North American and Eurasian. The Eurasian lineage is further split into three sublineages: Old-Eurasian, European–Asian and Far-Eastern [37]. In this study H7N3 viruses belong to the European–Asian sublineage, showing a close relationship with the H7 segment of subtypes, such as H7N7 from Georgia (2016), South Korea (2018), Mongolia (2019) and Japan (2019), and share a common ancestor with H7N3 viruses that were isolated in Mongolia in 2017 (Figure A1). It should be noted that the H7 HA genes of the Dagestan H7N3 viruses are genetically distinct from novel H7N3 reassortment, originating from the zoonotic H7N9 HPAIV, recently detected from poultry meat illegally imported from China into Japan in March 2018 [11], which belong to the Far-Eastern sublineage. To trace the evolutionary origin of the H7 HA gene segment of the Dagestan H7N3 isolates, we did not include the Far-Eastern sublineage in our phylogenetic analysis. During evolution, European–Asian and Far-Eastern sublineages started to evolve separately, becoming more and more different over time.

Recent outbreaks of low-pathogenicity avian influenza A(H7N3) virus of North American wild bird lineage in poultry confirmed that the virus can mutate to the highly pathogenic form in a very short time [38]. Thus, we assessed the threat of H7N3 viruses to human, poultry and wild birds in the Caspian region. In the present study we did not find genetic markers suggesting a highly pathogenic virus phenotype (amino acid substitutions in HA, NA, PB2, PB1-F2, NS1). In vivo experiments confirmed the viruses to be low pathogenic in chicken and mouse animal models. However, low pathogenic phenotypes of H7 AIV can spontaneously mutate to the HPAI by several different mechanisms, such as acquiring of basic amino acids to the hemagglutinin (HA) cleavage site to generate a multibasic cleavage site (MCS) in the H7 HA protein, causing widespread disease in poultry [38]. In addition, even a low pathogenicity avian influenza A(H7N3) virus, in combination with other pathogens, can cause mortality among ducks [15]. Thus, continued surveillance and pathotyping of H7 AIV in animal models and by sequencing is warranted wherever H7 AIV have been detected in wild birds.

Amino acid analysis showed that the receptor-binding sites in the H7 HA proteins indicated a preference for α2,3-linked sialic acid receptors, which are predominant in avian species. Although our study didn’t reveal any substitutions associated either with H7 adaptation to poultry or conferring an adaptation to mammalian hosts, the sporadic emergence of H7 HPAI is a cause for concern. Thousands of migratory birds share an ecologically diverse habitat in the Caspian Sea region, where migratory pathways from Africa and Asia intersect with those to and from Siberia [13]. And, as stated above, the pathogenic and zoonotic potential of H7 strains makes them a substantial economic and public health concern. Thus, the Caspian region may be underestimated as to its role in AIV dispersal and may be of great interest as a possible migratory stopover site where different strains of AIV can reassort. The ecological context is similar and potentially connected to regional “hot spots” for AIV reassortment in the Black and Azov Sea regions of Ukraine and the wetlands of Georgia [5,39]. Indeed, cladistically related gene segments were found in H7 AIV in mallard ducks in Ukraine in 2014 and Georgia 2016 (Figure A1).

In conclusion, H7N3 low pathogenic AIVs were identified in wild mallard ducks in the Caspian region of Russia. The findings of this study suggest that H7N3 viruses may frequently undergo reassortment and can be a natural recombinant that includes viral gene segments shared with both LPAIV and HPAIV. Thus, our results highlight the need for continuous and intensive surveillance of avian influenza in this region, with very active intersection of wild bird flyways suggestive of the exchange of AIV genes in a multi-host reservoir over a wide swath of Eurasia.

## Data Availability

Sequence accession numbers in GISAID (global initiative on sharing avian flu data) are as follows: EPI_ISL_331289 for A/mallard/Dagestan/1050/2018; and EPI_ISL_331290 for A/mallard/Dagestan/1051/2018.

## Supplementary Materials

The following are available online:https://www.mdpi.com/article/10.3390/microorganisms9040864/s1; Figure S1: HA_H3 numbering; Figure S2: Nucleotide sequences percent identities; Figure S3: ML phylogenetic trees of the PB2, PB1, PA, HA, NP, NA, MP, and NS genes of the H7N3 AIV; Figure S4: Root-to-tip regression analysis; Table S1: Results of HI assay; Table S2: HA cleavage site and RBS; Table S3: NCBI & GISAID_BLAST RESULTS; Additional Information 1: Amino acid positions in FLU07-1050 and FLU08-1051 H7N3 viruses.

## Appendix A

**Table A1:** The substitution models used in the molecular phylogenetic analyses by the maximum likelihood (ML) method.

No	Segment	Best-Fit Model of Substitution According to BIC	Input Data
1	PB2	GTR + F + G4	131 sequences with 2280 nt sites
2	PB1	TIM + F + I + G4	136 sequences with 2274 nt sites
3	PA	TVM + F + G4	119 sequences with 2151 nt sites
4	HA	TVM + F + G4	104 sequences with 1683 nt sites
5	NP	K3Pu + F + R2	105 sequences with 1497 nt sites
6	NA	TIM + F + I + G4	116 sequences with 1410 nt sites
7	MP	TIMe + I + G4	119 sequences with 982 nt sites
8	NS	HKY + F + R2	112 sequences with 838 nt sites
