# Supplementary material for: Biological Properties and Genetic Characterization of Novel Low Pathogenic H7N3 Avian Influenza Viruses Isolated from Mallard Ducks in the Caspian Region, Dagestan, Russia
Source: Microorganisms. 2021 Apr 17;9(4):864. doi: 10.3390/microorganisms9040864 (PMC8072542; doi:10.3390/microorganisms9040864)

Figure S4. Root-to-tip regression analysis. Plots of the root-to-tip genetic distance against sampling time are shown for phylogenies estimated from the 241 HA7 segment sequences alignments, sampled between 1999 and 2019.

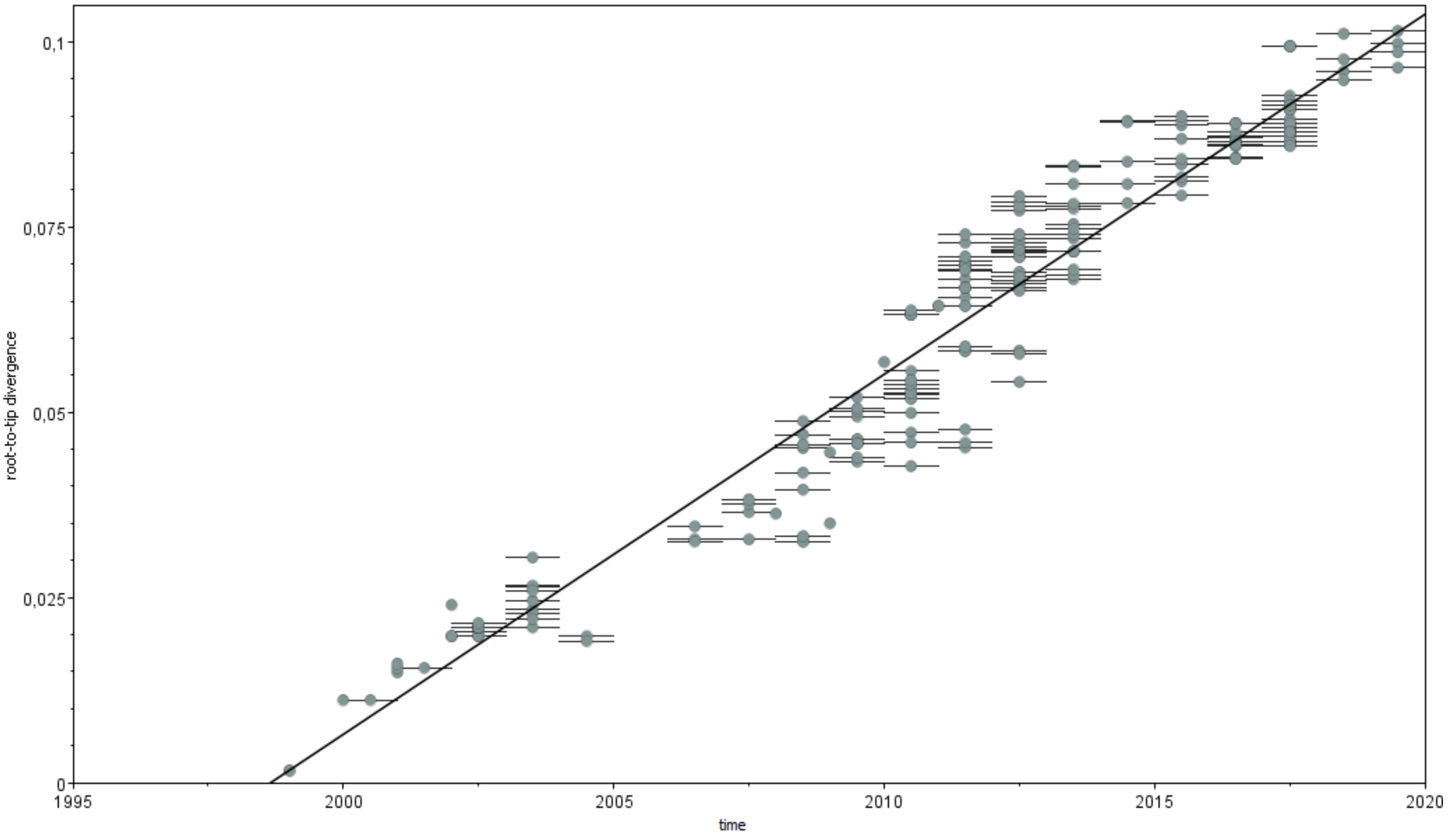

Supplement: Supplementary file 1 [file microorganisms-09-00864-s001.zip › microorganisms-1165576/REV_Supplementary Files/Figure S4. Root-to-tip regression analysis.pdf]
