# Supplementary material for: Biological Properties and Genetic Characterization of Novel Low Pathogenic H7N3 Avian Influenza Viruses Isolated from Mallard Ducks in the Caspian Region, Dagestan, Russia
Source: Microorganisms. 2021 Apr 17;9(4):864. doi: 10.3390/microorganisms9040864 (PMC8072542; doi:10.3390/microorganisms9040864)
Supplement: Supplementary file 1 [file microorganisms-09-00864-s001.zip › microorganisms-1165576/REV_Supplementary Files/REV_Additional Information_1_Amino acid position in FLU07-1050 and FLU08-1051 H73 viruses.pdf]

Additional Information (AI 1): Amino acid positions in FLU07-1050 and FLU08-1051 H7N3 viruses compared to mutations/motifs (reported below) previously described in various AIV subtypes reviewed by

Suttie A, Deng YM, Greenhill AR, Dussart P, Horwood PF, Karlsson EA. Inventory of molecular markers affecting biological characteristics of avian influenza A viruses. Virus Genes. 2019;55(6):739-768. doi:10.1007/s11262-019-01700-z)

Mänz B, Schwemmle M, Brunotte L. Adaptation of avian influenza A virus polymerase in mammals to overcome the host species barrier. *J Virol*. 2013;87(13):7200-7209. doi:10.1128/JVI.00980-13

HA (See also Table S2 and Figure S1)

- H3 numbering relative to A/Aichi/2/1968, H3N2:
  158N previously described, in HA of H5N1, as leading to increased virus binding to α2-6, increased transmission in guinea pig;
  160A previously described, in HA of H5N1, as leading to increased virus binding to α2-6, increased transmission in guinea pig;
  393E previously described, in HA of **H7N9**, as leading to increased pH of fusion, decreased HA stability, decreased virulence in mice.

NA

- No mutations previously reported by Suttie et al. (2019) for N3 were observed.
- No amino acid deletion of the stalk region of the N3 associated with H7 virulence was detected.

MP

- Numbering relative to allignment with A/goose/Guandong/1/96:
  30D, 215A previously described, in M1 of H5N1, as leading to increased virulence in mice;
  43M previously described, in M1 of H5N1, as leading to increased virulence in mice, chickens, ducks.

PB1

- Numbering relative to allignment with A/goose/Guandong/1/96:
  3V previously described, in PB1 of H5N1, as leading to increased polymerase activity and viral replication in avian and mammalian cell lines.

PB2

- Numbering relative to allignment with A/goose/Guandong/1/96:

  389R previously described, in PB2 of **H7N9**, as leading to increased polymerase activity and viral replication in mammalian cells;

  598T previously described, in PB2 of **H7N9**, as leading to increased polymerase activity and viral replication in mammalian cells and increased virulence in mice;

  89V, 309D and **89V, 309D, 339K, 477G, 495V, 627E, 676T** aa (motifs) in patterns previously described, in PB2 of H5N1, as leading to altered viral polymerase activity in mammalian cells and virulence in mice.

PA

- <u>Numbering relative to allignment with A/goose/Guandong/1/96:</u>
  37A previously described, in PA of **H7N9**, as leading to increased polymerase activity in mammalian cells;
  190S previously described, in PA of **H7N3**, as leading to decreased virulence in mouse;
  383D previously described, in PA of H5N1, as leading to increased polymerase activity in mammalian cells and avian cell lines;
  409S previously described, in PA of **H7N9,** as leading to increased polymerase activity in mammalian cells and replication in mammalian cell lines.
  There are no mutations associated with mammalian adaptation (336M, 552S and 615N) (Mänze et al., 2013).

NP

- <u>Numbering relative to allignment with A/goose/Guandong/1/96:</u>
  105V previously described, in NP of H5N1, as leading to increased virulence in chicken;
  184K previously described, in NP of H5N1, as leading to increased replication in avian cells and virulence in chickens, enhanced IFN response;
  198R previously described, in NP of H5N1 viruses, as leading to decreased polymerase activity in mammalian cells;
  There are no mutations associated with mammalian adaptation (16D, 283P, 313Y and 357K) (Mänze et al., 2013).

NS

- <u>Numbering relative to allignment with A/goose/Guandong/1/96:</u>

  42S previously described, in NS1 of H5N1, as leading to increased virulence and decreased antiviral response in mice;

  106M previously described, in NS1 of H1N1 with all internal genes from **H7N9**, as leading to increased viral replication in mammalian cells and virulence in mice;

  138F previously described, in NS1 of H5N1, as leading to increased replication in mammalian cells, decreased interferon response;

  149A previously described, in NS1 of H5N1, as leading to increased virulence and decreased interferon response in chicken;

  205S previously described, in NS1 of H5N1, as leading to decreased antiviral response in ferret;

  103F,106M motif previously described, in NS1 of H5N1, as leading to increased virulence in mouse;

  55E, 66E, 138F motif previously described, in NS1of H5N1, as leading to increased mammalian cell replication, decreased response to interferon.
