# Supplementary material for: Biological Properties and Genetic Characterization of Novel Low Pathogenic H7N3 Avian Influenza Viruses Isolated from Mallard Ducks in the Caspian Region, Dagestan, Russia
Source: Microorganisms. 2021 Apr 17;9(4):864. doi: 10.3390/microorganisms9040864 (PMC8072542; doi:10.3390/microorganisms9040864)
Supplement: Supplementary file 1 [file microorganisms-09-00864-s001.zip › microorganisms-1165576/REV_Supplementary Files/REV_Figure S1_HA_H3 numbering.pdf]

**Figure S1.** Amino acid sequence alignment (H3 numbering) of HA protein from H7N3 AIVs A/mallard/Dagestan/1050/2018 (HA-07) and A/mallard/Dagestan/1051/2018 (HA-08)

```
               10        20        30        40        50        60        70        80        90       100
       ....|....|....|....|....|....|....|....|....|....|....|....|....|....|....|....|....|....|....|....|
HA-07_
MNTQILVLALVAIIPTNADKICLGHHAVSNGTKVNTLTERGVEVVNATETVERTNVPRICSKGKRTVDLGQCGLLGTITGPPQCDQFLEFSADLIVERRE
HA-08_
MNTQILVLALVAIIPTNADKICLGHHAVSNGTKVNTLTERGVEVVNATETVERTNVPRICSKGKRTVDLGQCGLLGTITGPPQCDQFLEFSADLIVERRE
A/Aichi/2/68 H3N2
~~~~~~~~QDLPGNDNSTATLCLGHHAVPNGTLVKTITDDQIEVTNATELVQSSSTGKICNNPHRILDGIDCTLIDALLGDPHCDVFQNETWDLFVERSK

              110       120       130       140       150       160       170       180       190       200
       ....|....|....|....|....|....|....|....|....|....|....|....|....|....|....|....|....|....|....|....|
HA-07_
GSDVCYPGKFVNEEALRQILRESGGIDKETMGFTYSGIRTNGATSACKR~SGSSFYAEMKWLLSNTDNAAFPQMTKSYKNTRKDPALIIWGIHHSGSTTE
HA-08_
GSDVCYPGKFVNEEALRQILRESGGIDKETMGFTYSGIRTNGATSACRR~SGSSFYAEMKWLLSNTDNAAFPQMTKSYKNTRKDPALIIWGIHHSGSTTE
A/Aichi/2/68 H3N2
AFSNCYPYDVPDYASLRSLVASSGTLEFITEGFTWTGVTQNGGSNACKRGPGSGFFSRLNWLTK~~SGSTYPVLNVTMPNNDNFDKLYIWGIHHPSTNQE

              210       220       230       240       250       260       270       280       290       300
       ....|....|....|....|....|....|....|....|....|....|....|....|....|....|....|....|....|....|....|....|
HA-07_
QTKLYGSGNKLITVGSSNYQQSFVPSPGARPQVNGQSGRIDFHWLMLNPNDTVTFSFNGAFIAPDRASFLR~GKSMGIQSGVQVDASCEGDCYHNGGTII
HA-08_
QTKLYGSGNKLITVGSSNYQQSFVPSPGARPQVNGQSGRIDFHWLMLNPNDTVTFSFNGAFIAPDRASFLR~GKSMGIQSGVQVDASCEGDCYHNGGTII
A/Aichi/2/68 H3N2
QTSLYVQASGRVTVSTRRSQQTIIPNIGSRPWVRGLSSRISIYWTIVKPGDVLVINSNGNLIAPRGYFKMRTGKSSIMRSDAPIDT~CISECITPNGSIP

              310       320       330       340       350       360       370       380       390       400
       ....|....|....|....|....|....|....|....|....|....|....|....|....|....|....|....|....|....|....|....|
HA-07_
SNLPFQNINSRAVGKCPRYVKQESLMLATGMKNVPELPKGRGLFGAIAGFIENGWEGLIDGWYGFRHQNAQGEGTAADYKSTQSAIDQITGKLNRLIEKT
HA-08_
SNLPFQNINSRAVGKCPRYVKQESLMLATGMKNVPELPKGRGLFGAIAGFIENGWEGLIDGWYGFRHQNAQGEGTAADYKSTQSAIDQITGKLNRLIEKT
A/Aichi/2/68 H3N2
NDKPFQNVNKITYGACPKYVKQNTLKLATGMRNVPEK~QTRGLFGAIAGFIENGWEGMIDGWYGFRHQNSEGTGQAADLKSTQAAIDQINGKLNRVIEKT
```

```
                410       420       430       440       450       460       470       480       490       500
       ....|....|....|....|....|....|....|....|....|....|....|....|....|....|....|....|....|....|....|....|
HA-07_
NQQFELIDNEFTEVEKQIGNVINWTRDAMTEVWSYNAELLVAMENQHTIDLADSEMNKLYERVKRQLRENAEEDGTGCFEIFHKCDDDCMASIRNNTYDH
HA-08_
NQQFELIDNEFTEVEKQIGNVINWTRDAMTEVWSYNAELLVAMENQHTIDLADSEMNKLYERVKRQLRENAEEDGTGCFEIFHKCDDDCMASIRNNTYDH
A/Aichi/2/68 H3N2
NEKFHQIEKEFSEVEGRIQDLEKYVEDTKIDLWSYNAELLVALENQHTIDLTDSEMNKLFEKTRRQLRENAEEMGNGCFKIYHKCDNACIESIRNGTYDH

                                510       520       530       540       550       560
                       ....|....|....|....|....|....|....|....|....|....|....|....|...
HA-07_                 SKYREEAMQNRIQINPVKLSSGYKDVILWFSFGASCFILLAIAMGLVFICVKNGNMRCTICI*
HA-08_                 SKYREEAMQNRIQINPVKLSSGYKDVILWFSFGASCFILLAIAMGLVFICVKNGNMRCTICI*
A/Aichi/2/68 H3N2      DVYRDEALNNRFQIKGVELKSGYKDWILWISFAISCFLLCVVLLGFIMWACQRGNIRCNICI*
```

Legend:
Receptor binding site: red boxes
Clivage site: black box
Glycosylation sites: green boxes
