# Supplementary material for: Biological Properties and Genetic Characterization of Novel Low Pathogenic H7N3 Avian Influenza Viruses Isolated from Mallard Ducks in the Caspian Region, Dagestan, Russia
Source: Microorganisms. 2021 Apr 17;9(4):864. doi: 10.3390/microorganisms9040864 (PMC8072542; doi:10.3390/microorganisms9040864)
Supplement: Supplementary file 1 [file microorganisms-09-00864-s001.zip › microorganisms-1165576/REV_Supplementary Files/Table S1_ Results of HI assay.pdf]

Table S1. Results of HI assay

**Virus**	**Sera**	
	A/mallard/Dagestan/1050/2018 (H7N3)	A/mallard/Dagestan/1051/2018(H7N3)
A/mallard/Dagestan/1050/2018(H7N3)	1/20	1/20
A/mallard/Dagestan/1051/2018(H7N3)	1/20	1/40
