# Supplementary material for: Biological Properties and Genetic Characterization of Novel Low Pathogenic H7N3 Avian Influenza Viruses Isolated from Mallard Ducks in the Caspian Region, Dagestan, Russia
Source: Microorganisms. 2021 Apr 17;9(4):864. doi: 10.3390/microorganisms9040864 (PMC8072542; doi:10.3390/microorganisms9040864)
Supplement: Supplementary file 1 [file microorganisms-09-00864-s001.zip › microorganisms-1165576/REV_Supplementary Files/Table S2_HA cleavage site and RBS.pdf]

Table S2. Amino acids composition of the HA cleavage site and receptor-binding site of the HA gene of H7N3 strains.

	**HA cleavage**	**Receptor-binding site**						**Left edge of Receptor-binding site**	**Right edge of Receptor-binding site**
H7 position:	333-342	106	162	192	199	202	203	229-238	142-146
A/mallard/Dagestan/1050/2018	PELPKGRGLF	Y	L	H	E	K	L	RPQVNGQSGR	GATSA
A/mallard/Dagestan/1051/2018	PELPKGRGLF	Y	L	H	E	K	L	RPQVNGQSGR	GATSA
