# Supplementary figures and images for: Biological Properties and Genetic Characterization of Novel Low Pathogenic H7N3 Avian Influenza Viruses Isolated from Mallard Ducks in the Caspian Region, Dagestan, Russia
Source: Microorganisms. 2021 Apr 17;9(4):864. doi: 10.3390/microorganisms9040864 (PMC8072542; doi:10.3390/microorganisms9040864)

**Figure S2.** Levels of sequence identity between the FLU07-1050 and FLU08-1051 H7N3 viruses.

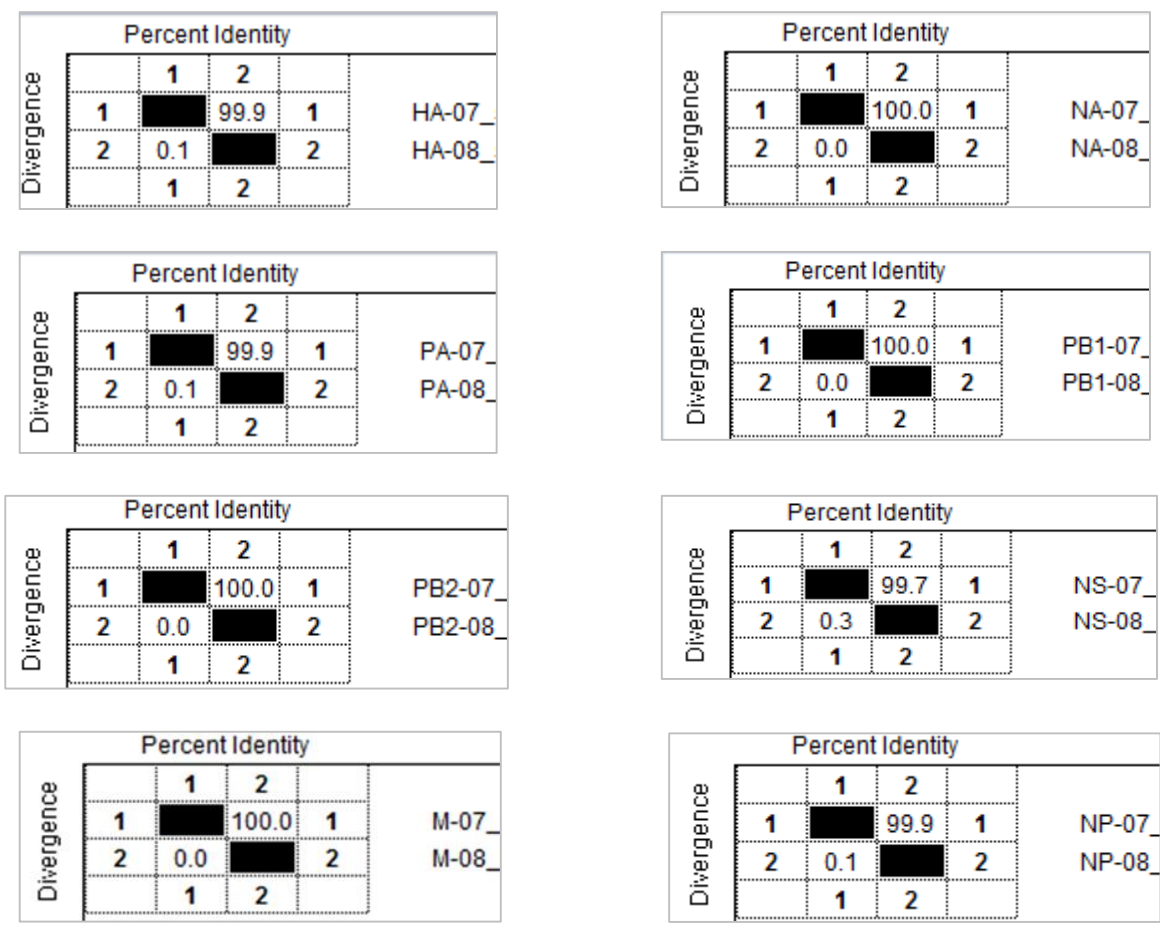

Supplement: Supplementary file 1 [file microorganisms-09-00864-s001.zip › microorganisms-1165576/REV_Supplementary Files/REV_Figure S2_Nucleotide sequences percent identities.pdf]
